# Increased Serum NSE and S100B Indicate Neuronal and Glial Alterations in Subjects Under 71 Years With Mild Neurocognitive Disorder/Mild Cognitive Impairment

**DOI:** 10.3389/fncel.2022.788150

**Published:** 2022-07-14

**Authors:** Maryna Polyakova, Karsten Mueller, Katrin Arelin, Leonie Lampe, Francisca S. Rodriguez, Tobias Luck, Jürgen Kratzsch, Karl-Titus Hoffmann, Steffi Riedel-Heller, Arno Villringer, Peter Schoenknecht, Matthias L. Schroeter

**Affiliations:** ^1^Max Planck Institute for Human Cognitive and Brain Sciences, Leipzig, Germany; ^2^Clinic for Cognitive Neurology, University of Leipzig, Leipzig, Germany; ^3^LIFE–Leipzig Research Center for Civilization Diseases, Leipzig University, Leipzig, Germany; ^4^University Clinic for Psychiatry and Psychotherapy, Leipzig University, Leipzig, Germany; ^5^Research Group Psychosocial Epidemiology and Public Health, German Center for Neurodegenerative Diseases (DZNE), Greifswald, Germany; ^6^Faculty of Applied Social Sciences, University of Applied Sciences Erfurt, Erfurt, Germany; ^7^Institute of Laboratory Medicine, Clinical Chemistry and Molecular Diagnostics, Leipzig University, Leipzig, Germany; ^8^Institute of Neuroradiology, University Clinic, Leipzig, Germany; ^9^Institute of Social Medicine, Occupational Health and Public Health (ISAP), Leipzig University, Leipzig, Germany; ^10^Department of Psychiatry and Psychotherapy, University Affiliated Hospital Arnsdorf, Technical University of Dresden, Dresden, Germany

**Keywords:** mild cognitive impairment, white matter hyperintensities, S100B, neuron-specific enolase, NSE, Brain-Derived Neurotrophic Factor, BDNF

## Abstract

**Background:**

Mild cognitive impairment (MCI) is considered a pre-stage of different dementia syndromes. Despite diagnostic criteria refined by DSM-5 and a new term for MCI – “mild neurocognitive disorder” (mild NCD) – this diagnosis is still based on clinical criteria.

**Methods:**

To link mild NCD to the underlying pathophysiology we assessed the degree of white matter hyperintensities (WMH) in the brain and peripheral biomarkers for neuronal integrity (neuron-specific enolase, NSE), plasticity (brain-derived neurotrophic factor, BDNF), and glial function (S100B) in 158 community-dwelling subjects with mild NCD and 82 healthy controls. All participants (63–79 years old) were selected from the Leipzig-population-based study of adults (LIFE).

**Results:**

Serum S100B levels were increased in mild NCD in comparison to controls (*p* = 0.007*)*. Serum NSE levels were also increased but remained non-significant after Bonferroni-Holm correction *(p* = 0.04). Furthermore, age by group interaction was significant for S100B. In an age-stratified sub-analysis, NSE and S100B were higher in younger subjects with mild NCD below 71 years of age. Some effects were inconsistent after controlling for potentially confounding factors. The discriminatory power of the two biomarkers NSE and S100B was insufficient to establish a pathologic threshold for mild NCD. In subjects with mild NCD, WMH load correlated with serum NSE levels (r = 0.20, *p* = 0.01), independently of age.

**Conclusion:**

Our findings might indicate the presence of neuronal (NSE) and glial (S100B) injury in mild NCD. Future studies need to investigate whether younger subjects with mild NCD with increased biomarker levels are at risk of developing major NCD.

## Introduction

Mild cognitive impairment (MCI) is a state of impaired cognition with preserved independence in daily living. It is regarded as a pre-stage of different neurodegenerative dementia syndromes, its amnestic subtype in particular for Alzheimer's disease (AD) (Schroeter et al., 2009a). The fifth edition of the Diagnostic and Statistical Manual of Mental Disorders (DSM-5), introduced a new term for MCI. The authors suggested naming it mild neurocognitive disorder (NCD) in contrast to major NCD, which replaced the term dementia (APA, 2013). Attempting to reflect neurobiology, the DSM-5 was expected to include biomarkers in the diagnostic criteria for dementia and its pre-stages (Carroll, 2013; Moller et al., 2015). Nevertheless, the evidence on molecular biomarkers of mild NCD was insufficient, and this diagnosis remained purely clinical instead (APA, 2013; Moller et al., 2015).

Searching for new evidence on biomarkers for mild NCD, we systematically searched for existing meta-analyses and compared here serum levels of three well-established biomarkers for neuropsychiatric diseases, namely the neuronal marker neuron-specific enolase (NSE), the plasticity marker brain-derived neurotrophic factor (BDNF), and the glial marker S100B in subjects with mild NCD and healthy volunteers. While these proteins are relatively well-studied in other neuropsychiatric diseases, in MCI their role was not clarified, inconclusive or even absent so far.

As an important pathoetiological factor, we assessed white matter hyperintensities (WMH) - a magnetic resonance imaging (MRI) marker for common age-related cerebral small vessel disease (SVD) (Kynast et al., 2018; Hamilton et al., 2021). WMHs are associated with conversion from subjective cognitive impairment or MCI to dementia (Gorelick et al., 2011; Benedictus et al., 2015). WMHs may lead to vascular dementia or, in the nomenclature of the DSM-5, vascular NCD (Schroeter et al., 2007; Kim et al., 2008; Quinque et al., 2012; Wardlaw et al., 2013). The American Heart Association included neuroimaging evidence for SVD or stroke in diagnostic criteria of vascular cognitive impairment (Gorelick et al., 2011).

S100B is a Ca-binding protein involved in protein phosphorylation, energy metabolism, and cytoskeletal organization of the cell. Being mainly expressed by astrocytes, and subpopulations of oligodendrocytes (Donato et al., 2009), S100B is regarded as a glial marker protein. S100B has been consistently shown to be involved in psychiatric diseases such as mood disorders and schizophrenia (Schroeter et al., 2008, 2009b, 2010, 2011, 2013, 2014; Schroeter and Steiner, 2009; Polyakova et al., 2015a). The chronic overexpression of S100B was discussed to be part of the neurodegenerative cytokine cycle in AD (Griffin et al., 1998; Griffin and Barger, 2010; Ferguson et al., 2017). Studies reported increased levels of S100B in cerebrospinal fluid (CSF) in mild/moderate AD compared to severe AD and healthy controls (Peskind et al., 2001), or in AD if compared with healthy controls (Petzold et al., 2003; Bellaver et al., 2021). Levada and Trailin (2012) found increased serum S100B in vascular dementia compared to its pre-stage, vascular MCI. A recent meta-analysis showed a high effect size (SMD 2.9) for S100B increment in serum of patients with AD (Bellaver et al., 2021). Moreover, S100B has been discussed to be involved in other neurodegenerative diseases (Steiner et al., 2011; Gmitterova et al., 2020; Langeh and Singh, 2021).

NSE is an intracellular glycolytic enzyme, largely expressed in neurons (Isgro et al., 2015), entirely utilized within the cell body, and not actively secreted (Steiner et al., 2006). Following damage to neuronal bodies or axons, NSE spreads into extracellular space and CSF (Lima et al., 2004), making it a perfect peripheral biomarker of neuronal damage. Elevated levels of serum NSE were consistently linked with cerebral ischemia, encephalitis, Parkinson's disease, etc. Previous findings in AD were mixed: no changes of NSE in serum or CSF (van den Doel et al., 1988; Parnetti et al., 1995) or increased NSE in CSF (Palumbo et al., 2008; Schmidt et al., 2014). To our knowledge, no study has examined serum NSE in MCI so far. A meta-analysis of NSE in Alzheimer's disease showed increased NSE levels in CSF of patients vs. controls, and comparable plasma or serum NSE levels (Olsson et al., 2016). Remarkably, increased serum NSE seems to be related to essential arterial hypertension (Gonzalez-Quevedo et al., 2011), the main risk factor for WMH related to MCI (Schroeter et al., 2005; Ungvari et al., 2021). WMH, in turn, is an independent risk factor for conversion from MCI to AD (Tokuchi et al., 2014; Tosto et al., 2014).

BDNF is responsible for activity-dependent synaptic plasticity and neurogenesis in the brain. It is expressed by neurons in the central and peripheral nervous system, in particular in the hippocampus (Polyakova et al., 2015b), besides peripheral tissues (Lommatzsch et al., 1999). Early studies on BDNF proposed a compensatory model where BDNF is increased in MCI and early AD, and profoundly exhausted in later stages of AD (Peng et al., 2005; Laske et al., 2006, 2007). However, later meta-analyses reported that BDNF levels were comparable to healthy subjects (Kim et al., 2017; Ng et al., 2019) or significantly decreased already in subjects with MCI (Xie et al., 2020). Unlike the large effect of BDNF reduction in depression (Polyakova et al., 2014), the most recent large-scale meta-analysis suggests that the effect size for BDNF decrement in MCI is small to medium (Ng et al., 2019; Xie et al., 2020). As the area under the curve (AUC) for discriminating MCI from HC was 0.57 (95% CI: 0.357–0.770, *p* = 0.558), it seems to have no diagnostic value for MCI screening (Xie et al., 2020).

In the current study, we assessed serum levels of biomarkers for glia, neurons, and plasticity – S100B, NSE, and BDNF – in subjects with mild NCD (diagnosed according to DSM-5) to test their biomarker eligibility. Based on previous research, we hypothesized increased levels of NSE and S100B in the serum of subjects with mild NCD. In an exploratory analysis, we evaluated the effects of BDNF, the correlation between serum markers and age, and the degree of WMHs in fast fluid-attenuated inversion recovery (FLAIR) MRI scans.

## Methods

### Subjects and Brain Imaging

Subjects with mild NCD and healthy controls were selected from the elder subpopulation (60–80 years) of the Leipzig Research Center for Civilization Diseases (LIFE-Adult), a population-based study (Loeffler et al., 2015). Only subjects with a full dataset (no missing data) were included, which resulted in an age range of participants from 63 to 79 years.

All subjects gave their written informed consent in accordance with the Declaration of Helsinki before participating in the study. The study was approved by the ethics board of the Medical Faculty of the University of Leipzig. At the moment of subject selection, the LIFE study database included 1,134 subjects over 60 years of age.

During the data collection phase, every subject filled out a questionnaire assessing subjective memory impairment, and underwent complex neuropsychological testing, clinical examination, blood sampling, and scanning with multimodal MRI. Psychiatric symptoms were evaluated by the Structured Psychiatric Interview for DSM-IV (SCID) and the Center for Epidemiological Studies Depression scale (CES-D). The degree of WMHs was evaluated with the four stages Fazekas scale (Fazekas et al., 1987) and in addition using the automated computer-based Topology-preserving Anatomy-Driven Segmentation algorithm (Lesion-TOADS) (Bazin and Pham, 2008; Shiee et al., 2010; Lampe et al., 2019). Fazekas score was assessed based on T2-weighted fluid-attenuated inversion recovery imaging (FLAIR). Three experienced neuroradiologists, blinded to diagnosis, classified the T2 FLAIR MRI scans into four Fazekas stages on the following criteria: Stage 0 (i.e., absence of punctate or confluent WMH), Stage 1 (punctate WMH), 2 (early confluent WMH), and 3 (large confluent WMH). We assessed medication intake using Anatomical Therapeutic Chemical (ATC) classification (https://www.whocc.no/atc/structure_and_principles/). Smoking status was assessed using the questionnaire and classified into “current smoker,” “non-smoker,” and “previous smoker.” Only subjects with a full dataset (no missing data in cognitive tests) were included, which resulted in an age range of participants from 63 to 79 years.

### Diagnostic Criteria

We used diagnostic criteria for mild NCD as implemented in DSM-5. They included the following criteria: (A) presence of subjective cognitive disturbance according to the self-assessment memory questionnaire; (B) objective cognitive decline 1–2 standard deviations (SD) below sex and age-adjusted norms in at least one of five cognitive domains (see below); (C) preserved activities of daily living according to the Instrumental Activities of Daily Living (IADL) questionnaire; (D) absence of delirium; and (E) absence of major psychiatric illness according to SCID and CES-D.

Cognitive testing was performed using the German version of the Consortium to Establish a Registry for AD (CERAD)-plus a test battery. Specific tests or subtests were assigned to each DSM-5 cognitive domain. In particular, results of the Trail-Making-Test (TMT) A were used to evaluate attention and the TMT ratio B/A to evaluate executive function. CERAD wordlist learning test was used for the evaluation of learning and memory. Boston naming test and verbal fluency test “animals” were used for evaluation of language domain. CERAD drawing figure test was used for the assessment of perceptual-motor function. The social cognition domain was not assessed during the subjects' selection. For each test individual participant's scores were compared to the normative score adjusted for age, sex and education. The normative values adjusted for sex, age, and education were obtained from the Basel memory clinic (www.memoryclinic.ch). A mean deviation from the norms was calculated for each cognitive domain when this domain was assessed with more than one test.

### Laboratory Procedures

Blood samples were collected from the subject's cubital vein in the morning after overnight fasting. Serum was prepared using the standard operating procedures. In brief, samples were left for 45 min for clotting, followed by a centrifugation step (10 min, 2750 g, 15°C). Samples were then filled in straws (CryoBioSytems IMV, France) by an automatic aliquoting system (DIVA, CryoBioSytems IMV, France). Thereafter serum samples were stored at -80°C. To minimize freeze-thaw cycles, samples were sorted in a cryogenic workbench (temperatures below -100°C) and automatically stored in tanks with a coolable top frame in the gas phase of liquid nitrogen [Askion, Germany (Loeffler et al., 2015)]. Storage time for all samples was no longer than 3 years.

S100B and NSE were measured with monoclonal 2-site immunoluminometric assays performed on the fully mechanized system LIAISON (Diasorin, Dietzenbach, Germany). The detection limit for the assays was 0.02 μg/l and 0.04 μg/l [described in detail elsewhere (Streitbuerger et al., 2012)]. BDNF was measured in serum with an Enzyme-Linked Immunosorbent Assay (ELISA), manufactured by R&D Systems [Wiesbaden, Germany; (Mueller et al., 2016)]. The sensitivity of the assay was 20 ng/L leading to a measuring range of 62.5 until 4,000 ng/L.

### Statistical Analysis

Statistical analyses were performed with RStudio Version 3.6.3 (R Team: RStudio, 2020). Complex assessments of the data and residuals' distributions were performed including visual assessment of the histograms, skewness and kurtosis of the data, Residuals vs. Fitted, Normal Q-Q, Scale-Location, and Residuals vs. Leverage plots. Due to deviations of biomarker data from the normal distributions data were log-transformed. Differences in demographic factors were assessed by independent *t*-tests or by chi-square tests. The group differences were assessed by linear modeling accounting for age and age-by-group interaction. Correlation analyses between serum and imaging markers were performed by calculating partial Spearman's correlation coefficients in the Ppcor package. Area under the curve analyses were performed using the “pROC” and “RandomForest” packages for R.

After observing interaction effects we performed a subsampling analysis with the median split according to age. The younger group was defined as subjects younger than the median of the entire sample, older age as the median and older. This analysis had an exploratory character.

The significance threshold of *p* < 0.05 was selected. We expected directed changes (increases) for NSE and S100B in mild NCD in comparison with control subjects, therefore one-tailed *p*-values were considered. For BDNF and correlation analyses two-tailed *p*-values were selected. We controlled for multiple comparisons with the Bonferroni-Holm correction, considering three markers as the family of tests. Generally, data are presented as mean and SD.

## Results

### Sample Characteristics

Clinical data and demographics are presented in Table 1. Our study sample comprised 158 subjects diagnosed with mild NCD and 82 healthy controls. The two samples did not statistically differ in sex, education, BMI, and the load of WMHs. However, subjects with mild NCD were significantly older than healthy subjects (*p* = 0.003). The effect size for the age difference was medium (Cohen's d = 0.45). Accordingly, statistical approaches were applied correcting for the factor age (see Methods section). As expected for such an elder population the majority of subjects took medication, for both HC and participants with MCI (76.8 and 91.3%; for details see Supplementary Table 1). Note that relevant behavioral abnormalities were absent in the MCI cohort (Supplementary Table 2).

**Table 1:** Demographical, clinical data, and serum levels of biomarkers in subjects with mild neurocognitive disorder and healthy control subjects.

	**Mild NCD**			**Healthy controls**		
	**All**	**Age <71 years**	**Age ≥71 years**	**All**	**Age <71 years**	**Age ≥71 years**
Number of subjects	158	60	98	82	47	35
Age (mean ± SD; years)	**71.7** ±3.8**	67.8 ± 2.0	74.0 ±2.5	**70.0** ±**4.1**	67.0 ±2.0	74.1 ±2.6
Sex (male/ female)	91/67	28/32	63/35	51/31	29/18	22/13
BMI (mean ± SD, missing kg/m^2^)	27.5 ±3.8	27.6 ±3.6	27.4 ± 4.0	28.1 ±4.6	28.2 ±5.2	27.9 ± 3.8
Education (<12/ >12 years)	114/44	48/12	66/32	58/24	33/14	25/10
Smoking status (smoker, non-smoker/previous smoker/missing data)	7/88/50/13	3/38/16/3	4/50/34/10	11/42/21/8	8/22/13/4	3/20/8/4
Fazekas score (0/1/2/3)	38/95/24/0	16/39/5/0	22/56/19/0	25/45/12/0	17/27/3/0	8/18/9/0
WMH load (mean ± SD, missing)	9.9 ± 12.7 (44)	6.5 ±7.0 (14)	12.1 ± 15.0 (30)	9.1 ± 10.7 (21)	6.2 ± 4.4 (10)	13.5± 15.4 (11)
BDNF (mean ± SD, missing, μg/L)	25.7 ±6.5	26.4 ±7.0	25.2 ± 6.2	25.2 ± 5.9	24.5 ± 4.6	26.2 ±7.2
NSE (mean ± SD, missing, μg/L)	**12.3** ±2.0 (2)*	**12.6** ±2.0**	12.1 ± 2.0	**11.9** ±2.1*	**11.5** ±2.0**	12.4 ±2.2
S100B (mean ± SD, missing, μg/L)	**0.08** ±0.04 (5)**	**0.08** ±0.03 (1)*	0.08 ± 0.04 (2)	**0.07** ±0.03 (2)**	**0.07** ±0.03*	0.11± 0.2 (2)

BDNF, brain-derived neurotrophic factor; BMI, body-mass index; HC, healthy controls; NCD, neurocognitive disorder; NSE, neuron-specific enolase; WMH, white matter hyperintensities; Test significance is labeled with the asterisks. *significant difference between subjects with mild NCD and HC at p <0.05;

^**^significant difference between subjects with mild NCD and HC at p < 0.01.

*Significant differences are shown with bold numbers*.

### Group Comparisons

**Figure 1:**
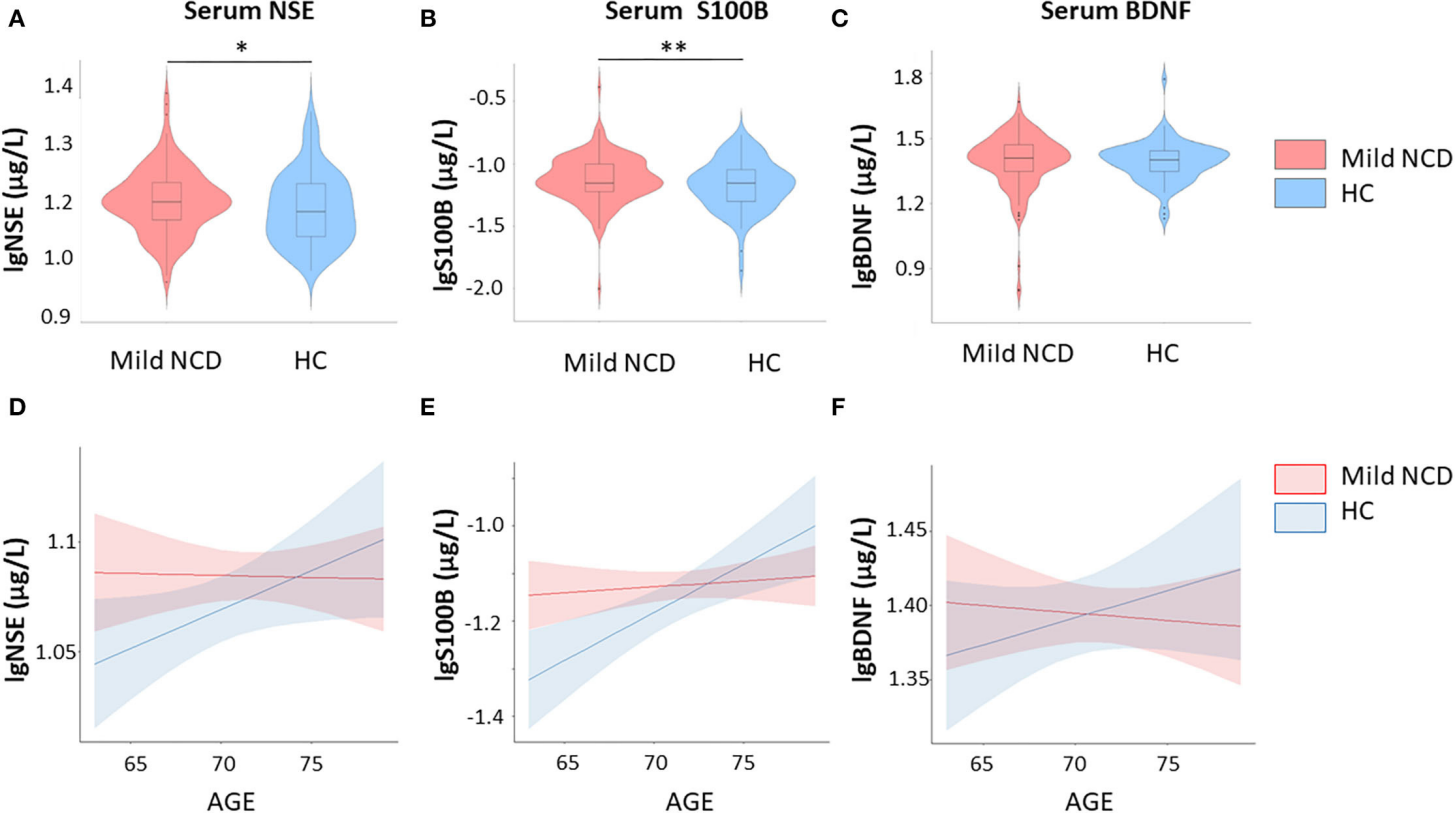
Group comparisons – S100B and neuron-specific enolase are increased in mild neurocognitive disorder. Furthermore, results of correlation between fluid biomarkers and age are shown. **(A)** Serum NSE levels in mild NCD vs. healthy controls; **(B)** Serum S100B levels in mild NSE vs. healthy controls; **(C)** Serum BDNF levels in mild NCD vs. healthy controls; **(D)** Correlation between serum NSE and age in mild NCD and HC; **(E)** Correlation between serum S100B and age in mild NCD and healthy controls; **(F)** Correlation between serum BDNF and age in mild NCD and healthy controls, BDNF Brain-Derived Neurotrophic Factor; NCD neurocognitive disorder; HC healthy controls; NSE neuron-specific enolase. * *p* < 0.05; ** *p* < 0.01.

Serum NSE, S100B, and BDNF were independent of each other in mild NCD and HC groups (Supplementary Table 4). As illustrated in Table 1 and Figure 1 we observed significant differences in glial and neuronal markers in the whole group analyses with higher values for serum S100B and NSE in mild NCD compared with healthy controls.

Multiple linear regression was used to predict serum S100B levels based on group, age, and group-by-age interaction. A significant regression equation was found [F (3, 230)=5.2, p = 0.001], with an R^2^ of 0.06. Group (β = −1.10, t (230) = −2.5, *p* = 0.009) and group-by-age interaction [β = 2.59, t (230) = 2.44 *p* = 0.016] explained significant amount of S100B variance, whereas age had no effect [β = 0.05, t (230) = 0.64, *p* = 0.52] (Figure 1). Cohen's d was 0.05 for the group effect. Both, group differences and interaction effects remained significant after correction for multiple comparisons.

The regression model for NSE [R^2^ = 0.03, F (3, 234) = 2.65, *p* = 0.05] revealed a significant effect for group, i.e., increased values in mild NCD compared to healthy controls (β = 0.29, t (230) = −1.75, *p* = 0.04), whereas age (β = −0.0006, t (230) = −0.39, *p* = 0.69) was not significant and interaction showed a trend (β = 0.004, t (230) = 1.67, *p* = 0.09). Cohen's d was 0.22 for the group effect. The group effect did not reach the significance threshold after Bonferroni-Holm correction.

For BDNF the regression model [R^2^ = 0.006, F (3, 236) = 0.50, *p* = 0.68] did not indicate significant effects for group [β = −0.32, t (230) = −1.16, *p* = 0.25), age (β = −0.006, t(230) = −0.97, *p* = 0.34), and respective interaction [β = 0.005, t (230) = 1.16, *p* = 0.25). Hence, serum BDNF levels did not differ significantly between MCI and healthy subjects.

### Reanalysis With Controlling for Confounding Factors

To exclude the effects of confounding factors we performed additional analyses, controlling for the effects of sex, BMI, and the smoking status on the biomarker levels. Across all analyses, the S100B group effects and group by age interaction remained significant. The differences in serum BDNF remained non-significant. Differences in serum NSE became non-significant after controlling for all BMI, smoking status, and sex. BMI was not significantly related to serum levels of S100B, NSE, or BDNF. Effects of sex were small but significant in all analyses. Smoking had a small but significant effect on NSE levels (beta = −0.02, *p* = 0.003).

The effects of medications, i.e., antidiabetics, antihypertensives, beta-blockers, calcium channel blockers, and agents acting on the renin-angiotensin system (that mirror cardiovascular and metabolic diseases too) on serum biomarker levels were non-significant in all analyses (data not shown) with the exception of significant effects of antidiabetic drugs on serum NSE levels (beta = −0.02, *p* = 0.04). The results for serum S100B remained significant in all analyses: S100B was increased in the mild NCD group, and the group-by-age interaction remained significant. The results for serum NSE remained significant at *p* < 0.05, but were not significant after the Bonferroni-Holm correction. Serum BDNF remained unchanged after controlling for medications.

We additionally controlled the analysis for all the factors in one model: age, sex, BMI, smoking status, antidiabetic, antihypertensive medications, beta-blockers, calcium channel blockers, and agents acting on the renin-angiotensin system. After this correction, the S100B effects also remained significant.

To assess the relationship between serum markers and cognitive testing we performed a partial correlation analysis between S100B, NSE, and BDNF and the participants' decline in five cognitive domains, corrected by age. The results of this analysis are depicted in Supplementary Table 3. Only S100B was negatively related to executive function (Supplementary Table 3).

### Subgroup Comparisons With Stratification for Subtype and Age

Our mild NCD sample included 136 subjects with single-domain impairment: amnestic MCI (*n* = 25), attention (*n* = 55), executive (*n* = 55), language (*n* = 3), AND visuo-construction (*n* = 1), as well as 22 subjects with multi-domain impairment: amnestic-multi-domain (*n* = 14) and non-amnestic-multi-domain (*n* = 8). There were no significant differences between the biomarker levels in these groups.

Due to significant interaction effects between group and age, we performed a subgroup analysis according to a median-split by age. The median age in the sample was 71 years. Subjects with mild NCD younger than 71 years had increased serum NSE levels in comparison to controls (*p* = 0.004), while in the older subgroup the NSE levels were comparable. Serum S100B was also higher in younger subjects with mild NCD (*p* = 0.025), but not in the older subgroup. The effect for NSE remained significant after correction for multiple comparisons, whereas the effect for S100B did not.

### Reanalysis With Controlling for Confounding Factors

In the subgroup analysis, when controlling for all covariates in the younger subgroup, the NSE difference remains significant at *p* < 0.05 (*p* = 0.045) and the S100B difference remains significant on the trend level only (*p* = 0.08). Note that for this analysis we control for twelve factors in the total sample size of 107 participants (mild NCD *n* = 60, HC *n* = 47). In the older subgroup results remain unchanged.

### Correlation Analyses

**Figure 2:**
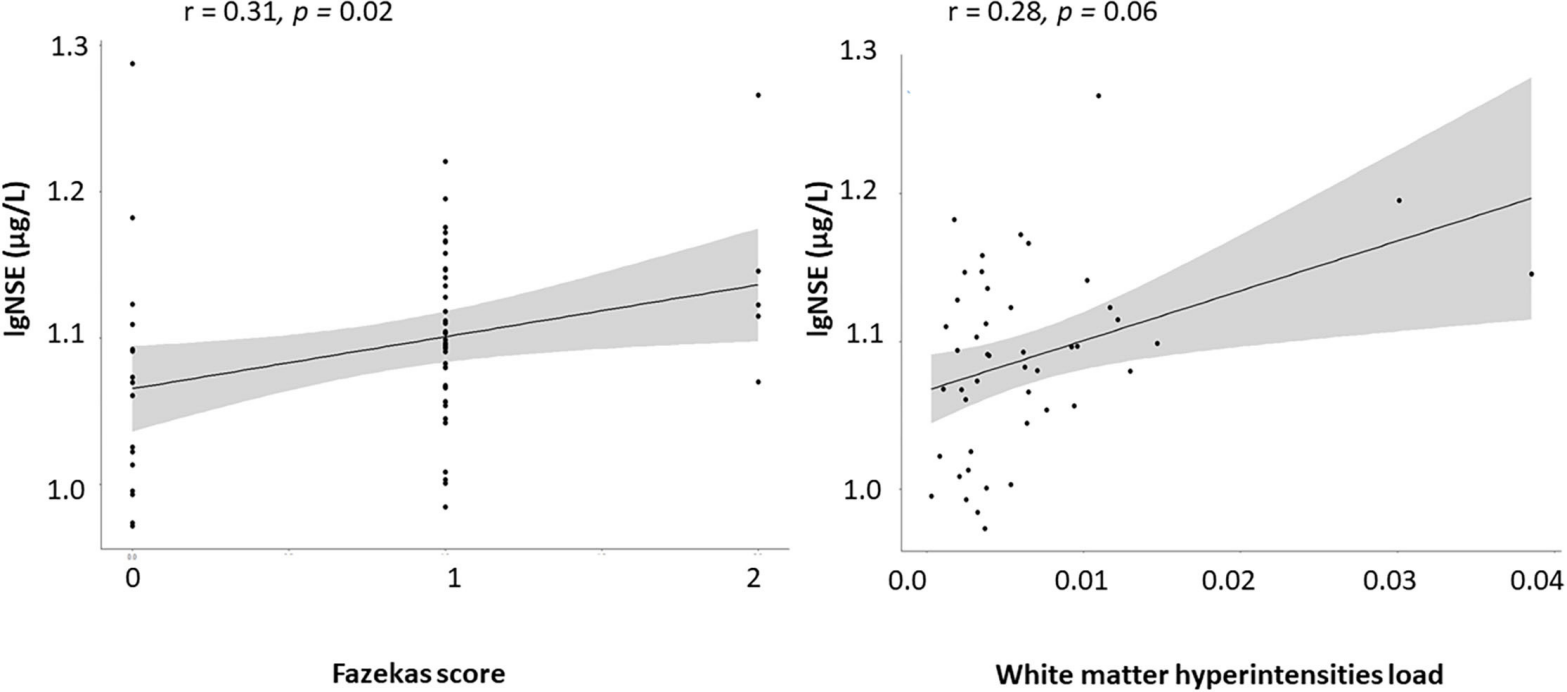
Correlation between WMH and serum NSE in the younger mild NCD subgroup.

Both white matter lesion measures – Fazekas score and WMH load – positively correlated with age in healthy subjects (r = 0.34/0.32, *p* = 0.002/0.01) and in subjects with mild NCD (r = 0.26/0.36, *p* = 0.001/ <0.001). Fazekas score correlated with serum NSE levels in subjects with mild NCD (r = 0.20, *p* = 0.01), independent of age, whereas WMH lesion load reached trend level only (r = 0.17, *p* = 0.06). In healthy subjects, S100B correlated positively with age (r = 0.32, *p* = 0.004) and BMI (r = 0.30, *p* = 0.009). The positive correlation of serum NSE with WMH measures was more pronounced in subjects with mild NCD younger than 71 years of age for WMH lesion load (r = 0.28, *p* = 0.06) and Fazekas score (r = 0.31, *p* = 0.02; Figure 2).

### Reanalysis With Controlling for Confounding Factors

To control for medication effects on the correlation between WMH and NSE in the mild NCD group, we performed partial correlation analyses controlling for age and additionally for cardiovascular medications taken by these subjects. The following medication groups were considered: antihypertensive medications, beta-blocking agents, calcium channel blockers, or agents acting on the renin-angiotensin system intake. In the whole mild NCD group, correlation effects between serum NSE and WMH load or Fazekas score became non-significant after correcting for antihypertensive medications, beta-blocking agents, calcium channel blockers, or agents acting on the renin-angiotensin system intake (data not shown).

After controlling for medication effects, the correlation between NSE and WMH parameters remained significant in mild NCD subjects under 71 years of age (NSE and WMH load r = 0.30, *p* = 0.04; NSE and Fazekas score 0.3, *p* = 0.03).

### Receiver Operating Characteristic Curve Analysis

**Figure 3:**
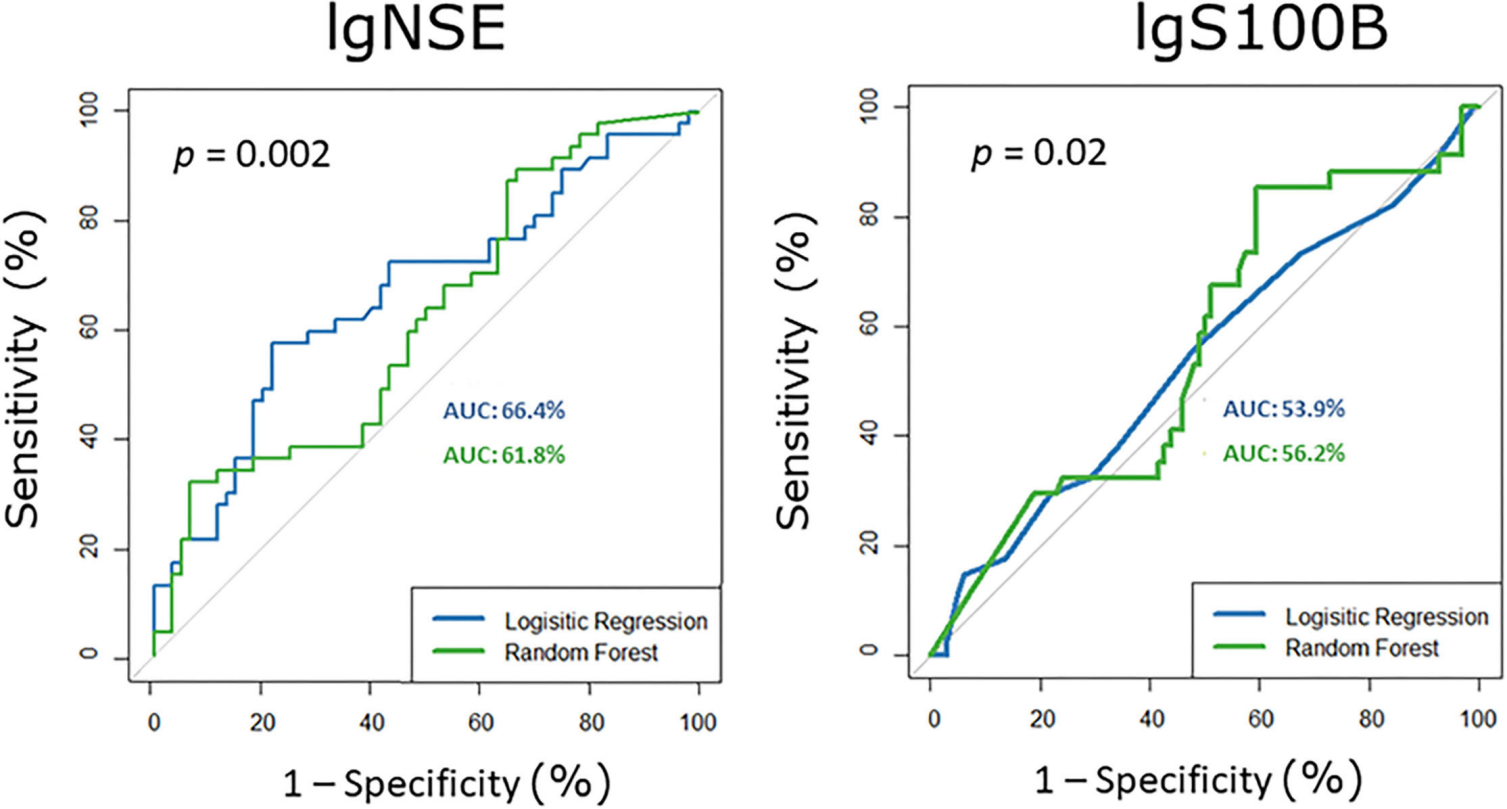
Receiver operating characteristic curves for serum S100B and NSE in the younger subgroup, i.e., participants with mild neurocognitive disorder vs. healthy controls. AUC area under the curve, NSE neuron-specific enolase.

The ROC analyses showed that serum NSE and S100B levels discriminate between healthy subjects and subjects with mild NCD in the younger subgroup (Figure 3) 6–16% better than chance (50%). For the older subgroup and for the pooled samples AUCs were not significantly different from 50%. For NSE the logistic regression approach outperformed random forest. For S100B random forest performed slightly better than the logistic regression.

## Discussion

Our current study provides evidence for increased levels of serum S100B and NSE in mild NCD. After controlling for possible confounding factors effects remained stable for S100B, but were inconsistent for NSE, possibly related to smoking and antidiabetic drugs. Both biomarkers were elevated in mild NCD subjects under 71 years of age in comparison with controls, which was not the case for subjects 71 years and older. Moreover, in subjects with mild NCD, the neuronal marker NSE correlated with the degree of WMH for both parameters. Fazekas scores and WMH load also correlated positively with age in subjects with mild NCD and healthy volunteers. In healthy participants, S100B correlated additionally with age and BMI. The discriminatory power of S100B and NSE was insufficient for establishing a pathological threshold for a biomarker of mild NCD.

### Elevated S100B Might Indicate Glial Involvement in Mild Neurocognitive Disorder

Our study is the first study of S100B in mild NCD. We observed increased S100B in the mild NCD group, in particular in younger subjects. An earlier study reported higher S100B in vascular dementia than vascular MCI in correlation with clinical impairments, although they did not compare values with healthy controls (Levada and Trailin, 2012). Serum S100B levels were also increased in patients with diabetes – a vascular risk factor – and cognitive decline compared to cognitively intact patients with diabetes (Yu et al., 2020). Serum S100B correlated with inflammatory markers in acute ischemic stroke (Beer et al., 2010) and has been shown to be an early marker of brain-blood barrier opening (Marchi et al., 2003). Higher S100B indicated reduced white matter integrity on diffusion tensor imaging (Streitbuerger et al., 2012). Serum S100B negatively correlated with fractional anisotropy and positively correlated with radial diffusivity in the white matter, in agreement with regional gene expression in the Allen human brain atlas. Furthermore, the study showed co-localization between S100B and the oligodendrocyte marker myelin basic protein on a histological level in the human corpus callosum. Taken together, the results suggested serum S100B as an indicator of oligodendrocytic function (Streitbuerger et al., 2012). In sum, elevated S100B in our sample might indicate inflammatory vascular brain damage and oligodendrocytic dysfunction.

Further potential mechanism of elevated S100B is its secretion in response to amyloid plaque formation. Activated astrocytes secret S100B to suppress amyloid-β aggregation *in vitro* (Langeh and Singh, 2021). In a clinical sample, CSF S100B was specifically associated with amyloid and tau-deposition status (Van Hulle et al., 2021). Christl et al. could not find differences between CSF S100B in patients with MCI related to AD and probable AD (Christl et al., 2019). According to Griffin and Barger (Griffin and Barger, 2010), immune responses by activated glia precede rather than follow AD pathology. In this view, increased S100B shall be expected in the early stages of AD. Our study indicates glial alterations in mild NCD, especially in younger subjects, which might be an early reaction to plaque formation.

The group and interaction effects remained stable after controlling for sex, BMI, smoking, and medications. The increased S100B observed in subjects under 71 years of age, but not in the older subgroup, might be attributed to those patients, who have an earlier onset of NCD. These effects should be investigated, as a predictor of conversion to major NCD, in future studies. Correlations of S100B with age that we observed have already been reported in the literature and are less relevant for the current work (Polyakova et al., 2015a).

### Elevated Neuron-Specific Enolase Might Indicate Neuronal Injury in Mild Neurocognitive Disorder

Increased levels of serum NSE may indicate neuronal injury in subjects with mild NCD, in particular those below 71 years of age. Two studies (Palumbo et al., 2008; Christl et al., 2019) – investigating NSE levels in amnestic MCI regarded as a pre-stage of AD (Christl et al., 2019) – did not find any differences between patients and controls in CSF. Christl et al. have investigated NSE levels in CSF in MCI-AD vs. AD patients and did not find evidence for different NSE levels (Christl et al., 2019). The majority of studies reported no differences between patients with AD and healthy controls in CSF (van den Doel et al., 1988; Parnetti et al., 1995; Nooijen et al., 1997) or serum (Lamour et al., 1988; Chaves et al., 2010).

At first glance, our results seem to be inconsistent with previous studies on AD and its pre-stage amnestic MCI. However, our sample is by definition very different from any AD or amnestic MCI sample. Here, we defined mild NCD based on DSM-5 criteria, including, besides its amnestic subtype (corresponding to amnestic MCI), impairments in memory in all other subtypes with a decline in other cognitive domains. Accordingly, our study considers besides Alzheimer-related NCD also other NCD types independent from etiological assumptions. Although our participants were not explicitly screened for biomarkers of AD, they mainly did not show the amnestic subtype of MCI regarded as a risk state for AD (Schroeter et al., 2009a). Rather, attention and executive function were mainly affected in our cohort, where it is well-known that vascular cognitive impairment such as in SVD is associated with cognitive impairment in these domains (Schroeter et al., 2007; Quinque et al., 2012; Kynast et al., 2018; Ungvari et al., 2021; Schroeter, 2022). Accordingly, our cohort reflects other or mixed etiologies compared with the former studies of AD. Note, moreover, that our study included a large number of subjects.

In sum, elevated serum NSE reflects higher levels of neuronal damage in (especially younger) subjects with mild NCD. The half-life of NSE in human serum is 24–30 h (Johnsson et al., 2000; Isgro et al., 2015). Hence, its serum levels reflect processes that occurred within this time period. Mean serum NSE levels in both groups were within the normative range. Accordingly, our data indicate only subtle brain damage in mild NCD. The correlation between NSE and Fazekas scores in the mild NCD group further points to an ongoing process (rather than a scar) or “a sign of the salient brain damage” as suggested by Gonzalez-Quevedo et al. (2011).

### Group-by-Age Interaction: NSE and S100B Changes Were Prominent in Subjects Younger Than 71 Years

Mild NCD is a heterogeneous category, where patients can progress to dementia, remain stable or even improve cognition. In our study younger subjects with mild NCD showed increased biomarker levels as compared to healthy controls, while the older group did not. We argue that these biomarkers should be investigated as predictors of progression to major NCD in longitudinal studies.

### Serum Neuron-Specific Enolase Might Be Related to White Matter Hyperintensities in Mild Neurocognitive Disorder

In correlation analyses, we observed a positive correlation between Fazekas scores and NSE. This finding was expected according to previous literature. The majority of WMHs have a vascular origin and accumulate with aging (Quinque et al., 2012; Aribisala et al., 2014; Shim et al., 2015). Remarkably, serum NSE positively correlated with WMHs' extent as quantified with Fazekas scores and quantitative WMH load in mild NCD. While in older subjects with mild NCD Fazekas scores correlated positively with age (similarly to healthy subjects), in the younger subgroup they instead correlated with NSE. The significant correlation between NSE and white matter lesions in the mild NCD group supports the relevance of vascular etiology in our cohort as already discussed.

In MCI a positive correlation between Fazekas scores and NSE has not been reported to our knowledge yet. Such correlation was found in patients with arterial hypertension (Gonzalez-Quevedo et al., 2011), the main risk factor for WMHs (Schroeter et al., 2005). In the study of Gonzalez-Quevedo both WMHs and increased NSE were related to the severity of arterial hypertension (Gonzalez-Quevedo et al., 2011). In this study, we used antihypertensive medications to indirectly control for the effects of hypertension. The correlation effects between WMH and serum NSE remained stable only in the younger mild NCD subgroup, while in the total mild NCD sample these correlations became non-significant. Our results indicate the involvement of vascular factors in the younger mild NCD subgroup; this association should be investigated in the future.

Two other studies explored WMH and NSE after open-heart surgery and reported conflicting findings. Stolz et al. did not find any association between NSE levels and new diffusion-weighted MRI hyperintensities after aortic valve replacement (Stolz et al., 2004). Steinberg et al. reported an increased number of WMH and NSE levels following open-heart surgery (Steinberg et al., 1996). While these studies discussed the acute ischemic nature of WMH, our study is more related to the chronic condition.

One might conclude that a number of participants in our mild NCD group (especially younger ones) have subtle brain damage due to cerebrovascular disease. Accordingly, serum NSE might be used as a peripheral biomarker for WMHs in mild (“vascular”) NCD in the future. From a histopathological point of view, WMH represents heterogeneous pathology, with demyelination, axonal degeneration, and inflammatory perivascular changes (Hommet et al., 2011; Schmidt et al., 2011; Shim et al., 2015). Our exploratory finding needs further replication, in particular in studies exploring involved pathomechanisms and relating WMHs to myelinic biomarkers.

### No Effects of BDNF in Mild Neurocognitive Disorder

We did not observe evidence for alterations of serum BDNF in mild NCD. To date the most comprehensive meta-analysis has shown decreased serum BDNF in MCI – but with a rather small effect size (Hedges' g = – 0.296) – and presumably related mainly to the amnestic subtype as a risk state for AD, diagnosed according to Petersen criteria (Xie et al., 2020). Hence, according to power calculations for obtaining the effect size of 0.30 both groups would require 175 subjects per group. Although with the mild NCD group we were approaching this sample size, our healthy group was smaller. Secondly, in our original dataset, we could not control for the effects of exercise (Bus et al., 2011; Balietti et al., 2018). The effects of medication also could not be clearly elucidated, as our sample was naturalistic by nature and the majority of participants were taking multiple medications. For the future BDNF analyses, we would recommend including at least 175 subjects per group and systematically controlling for potentially confounding factors (Balietti et al., 2018).

### Limitations of the Study

A small age difference between mild NCD and healthy groups might be regarded as a limitation of our study. However, we controlled for this issue in our analyses by including age as a factor or correcting correlations for age. Fazekas score might be criticized as a rough measure for the quantification of WMHs. However, literature shows a high correlation between Fazekas scores and volumetric assessment of WMH (Gao et al., 2011). Moreover, we included quantitative WMH lesion load in the analyses and confirmed our results with this measure.

One limitation of biomarker studies, including the current study, concerns biomarker expression in various cell types, not exclusively neuronal or glial origin (Michetti et al., 2019). To our knowledge, the origin of serum markers is currently not possible to elucidate *in-vivo* in human subjects.

Several factors can have an effect on fluid biomarkers. Due to the naturalistic sample in our study, we included subjects with somatic comorbidities and taking various medications. To account for these confounding effects we controlled for such factors as smoking, BMI, and antidiabetic and antihypertensive medications in our sample. In the BDNF analysis, we did not account for such factors as physical activity. Nevertheless, we excluded subjects with major psychiatric disorders, known to have a potential impact on S100B and BDNF. This might be the reason for the absence of significant BDNF effects. Nevertheless, we consider our initial analysis of value for the community and further directions in research, in particular as our result is in agreement with a meta-analysis on that issue (Xie et al., 2020).

Mild cognitive impairment or mild NCD is a clinical syndrome, rather than a pathological diagnosis. Accordingly, one might question whether biomarkers, as investigated in our study, might be related to it. The aim of the DSM-5 was to improve the diagnosis for potential treatment. With its chosen conceptual approach, i.e., defining specific clinical profiles for diseases with different pathological underpinnings, such as AD (mainly memory impairment), behavioral variant frontotemporal dementia (mainly behavioral/executive impairment) or primary progressive aphasia (mainly language impairment), it even tried to connect clinical profiles with pathological diagnosis. To address this issue we have performed a clinical subtype-specific analysis, which yielded no significant results. Future more comprehensive studies might identify subtype-specific correlations and shall take into consideration also conversion to specific major NCD/dementia syndromes with respective biomarkers in a longitudinal design approach.

## Conclusion

Our study investigated biomarkers for plasticity, neurons, and glia in mild NCD. We found increased serum S100B and increased serum NSE in mild NCD, as well as a correlation between NSE and WMH as measured with Fazekas scores and quantitative WMH load in subjects with mild NCD. Elevated S100B might indicate glial activation, while serum NSE might indicate neuronal injury due to vascular origin in mild NCD. NSE might be investigated as an early biomarker for WMH formation in the future. The biomarkers did not differ significantly between clinical mild NCD subtypes and did not show a sufficient discriminatory power for establishing a pathological threshold. As a limitation, some results showed inconsistent effects after controlling for potentially confounding factors. Further studies shall relate biomarkers specifically to each cognitive domain and etiology in mild NCD, and involve larger samples to further investigate their potential for characterizing pre-stages of several dementia syndromes. Future studies shall investigate whether younger subjects with mild NCD with increased biomarker levels are at risk for the development of major NCD.

## Data Availability Statement

The data analyzed in this study is subject to the following licenses/restrictions: the data that support the findings of this study are available from Leipzig Center for Civilization Diseases (LIFE) but restrictions apply to the availability of these data, which were used under license for the current study, and so are not publicly available. Data are however available from the authors upon reasonable request and with permission of the Leipzig Center for Civilization Diseases (LIFE). Requests to access these datasets should be directed to MP, polyakova@cbs.mpg.de.

## Ethics Statement

The study was approved by the Ethics board of the Medical Faculty of the University of Leipzig. The patients/participants provided their written informed consent to participate in this study.

## Author Contributions

MP, PS, KM, and MS have designed the experiment and interpreted the results. MP has selected the subjects and performed the analysis. MP and MS drafted the manuscript and had the lead in the revision of the manuscript. KA, LL, FR, TL, JK, K-TH, SR-H, AV, and PS have contributed substantially to the data acquisition. All authors were involved in critical revision of the manuscript, gave final approval of the version to be published, and agreed to be accountable for all aspects of the work in ensuring that questions related to the accuracy or integrity of any part of the work are appropriately investigated and resolved.

## Funding

This study has been supported by the International Max Planck Research School on Neuroscience of Communication (IMPRS NeuroCom; MP), by LIFE–Leipzig Research Center for Civilization Diseases at the University of Leipzig–funded by means of the European Union, by the European Regional Development Fund (ERDF) and by means of the Free State of Saxony within the framework of the excellence initiative (KA, FR, TL, SR-H, AV, PS, and MS), by the German Consortium for Frontotemporal Lobar Degeneration, funded by the German Federal Ministry of Education and Research (MS), by the German Research Foundation (DFG, MS, SCHR 774/5-1), and by the eHealthSax Initiative of the Sächsische Aufbaubank (SAB).

## Conflict of Interest

The authors declare that the research was conducted in the absence of any commercial or financial relationships that could be construed as a potential conflict of interest.

## Publisher's Note

All claims expressed in this article are solely those of the authors and do not necessarily represent those of their affiliated organizations, or those of the publisher, the editors and the reviewers. Any product that may be evaluated in this article, or claim that may be made by its manufacturer, is not guaranteed or endorsed by the publisher.
