# Supplementary material for: Increased Serum NSE and S100B Indicate Neuronal and Glial Alterations in Subjects Under 71 Years With Mild Neurocognitive Disorder/Mild Cognitive Impairment
Source: Front Cell Neurosci. 2022 Jul 14;16:788150. doi: 10.3389/fncel.2022.788150 (PMC9329528; doi:10.3389/fncel.2022.788150)

**Supplementary Table1. Medication intake in mild NCD and healthy subjects.**

| **Medication group according to ATC classification** | MCI (n=158) | HC (n=82) |
|---|---|---|
| Cardiovascular system | 219 | 82 |
| Alimentary tract and metabolism | 61 | 36 |
| Musculo-skeletal system | 41 | 16 |
| Nervous system | 37 | 19 |
| Systemic hormonal preparations, excl. sex hormones and insulins | 30 | 13 |
| Respiratory system | 23 | 13 |
| Blood and blood forming organs | 39 | 12 |
| Genito urinary system and sex hormones | 33 | 12 |
| Various | 14 | 8 |
| Antineoplastic and immunomodulating agents | 2 | 4 |
| Dermatologicals | 13 | 3 |
| Sensory organs | 31 | 2 |
| Antiinfectives for systemic use | 3 | 2 |
| None | 19 | 10 |

X-squared =20.644, df =13, p-value =0.08; ATC - Anatomical Therapeutic Chemical, HC healthy controls, MCI mild cognitive impairment.

**Supplementary Table 2. Behavioral abnormalities in subjects with mild NCD.**

| **Type** | Mild NCD (n=158) | | |
|---|---|---|---|
| | present | absent | not possible to assess |
| Personality changes | 0 | 158 | 0 |
| Altered social bahavior | 1 | 157 | 0 |
| Apathy | 1 | 156 | 1 |

## Supplementary Table 3. Correlation between serum markers and the degree of cognitive impairment in mild NCD*.

| Cognitive domain | MCI (n=158) | | |
|---|---|---|---|
| | S100B (Rho, p-value) | NSE | BDNF |
| Memory impairment | *Rho=0.06; p*=0.46 | *Rho=-0.05; p*=0.58 | *Rho=-0.05; p*=0.55 |
| Attention impairment | Rho=0.10; *p*=0.24 | *Rho=-0.04; p*=0.67 | Rho=-0.09; p=0.27 |
| Executive function impairment | Rho=-0.19; p=0.02 | *Rho=0.01; p*=0.87 | Rho=0.05; p=0.55 |
| Visuoconstruction impairment | Rho=-0.03; p=0.72 | *Rho=0.08; p*=0.32 | Rho=0.15; p=0.07 |
| Language impairment | Rho=0.08; p=0.34 | *Rho=0.02; p*=0.81 | Rho=0.03; p=0.69 |

*Spearman partial correlation corrected for age.

## Supplementary Table 4. Correlation between serum markers in mild NCD and HC.

| | Age < 70 | | | | | |
|---|---|---|---|---|---|---|
| | Mild NCD | | | HC | | |
| | **NSE** | **S100B** | **BDNF** | **NSE** | **S100B** | **BDNF** |
| **NSE** | *1* | *Rho=-0.10, p=0.27* | *Rho=0.13, p=0.40* | *1* | *Rho=-0.15, p=0.33* | *Rho=-0.04, p=0.80* |
| **S100B** | *Rho=-0.10, p=0.27* | *1* | *Rho=-0.15, p=0.33* | *Rho=-0.15, p=0.33* | *1* | *Rho=0.11, p=0.49* |
| **BDNF** | *Rho=0.13, p=0.40* | *Rho=-0.15, p=0.33* | *1* | *Rho=-0.04, p=0.80* | *Rho=0.11, p=0.49* | *1* |
| | | | | | | |
| | **Age >70** | | | | | |
| | **Mild NCD** | | | **HC** | | |
| | **NSE** | **S100B** | **BDNF** | **NSE** | **S100B** | **BDNF** |
| **NSE** | *1* | *Rho=-0.10, p=0.27* | *Rho=-0.09, p=0.37* | *1* | *Rho=0.14, p=0.15* | *Rho=-0.03, p=0.85* |
| **S100B** | *Rho=-0.10, p=0.27* | *1* | *Rho=-0.10, p=0.27* | *Rho=0.14, p=0.15* | *1* | *Rho=-0.05, p=0.78* |
| **BDNF** | *Rho=-0.09, p=0.37* | *Rho = -0.10, p=0.27* | *1* | *Rho=-0.03, p=0.85* | *Rho=-0.05, p=0.78* | *1* |

**Supplementary Figure 1 Serum S100B in mild NCD and HC**

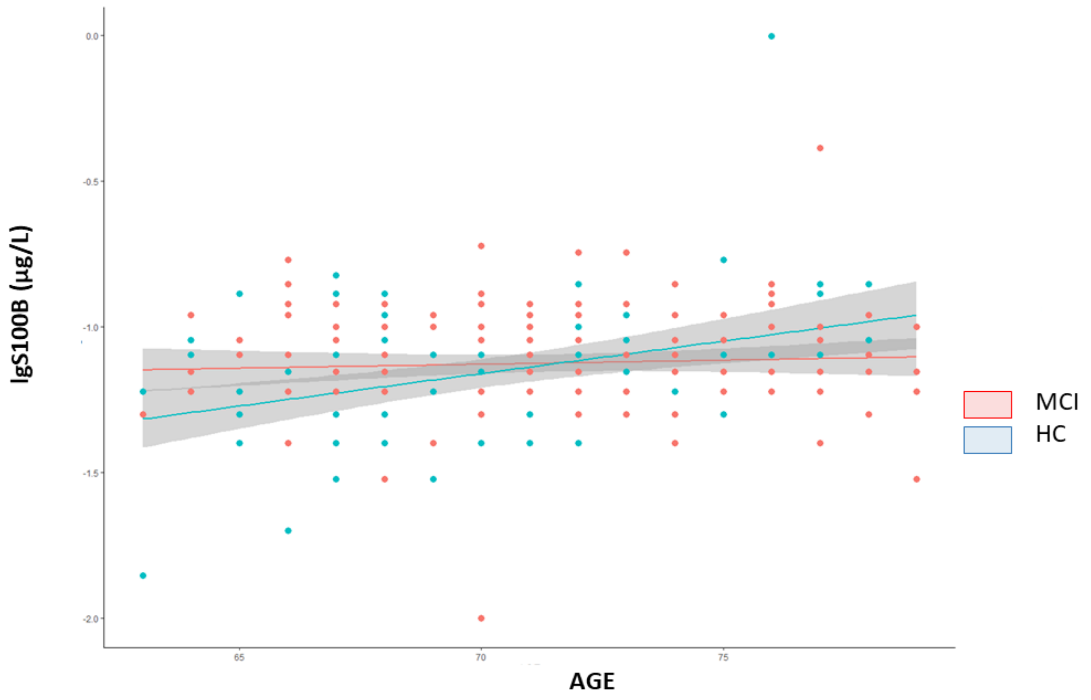


**Supplementary Figure 2 Serum NSE in mild NCD and HC**

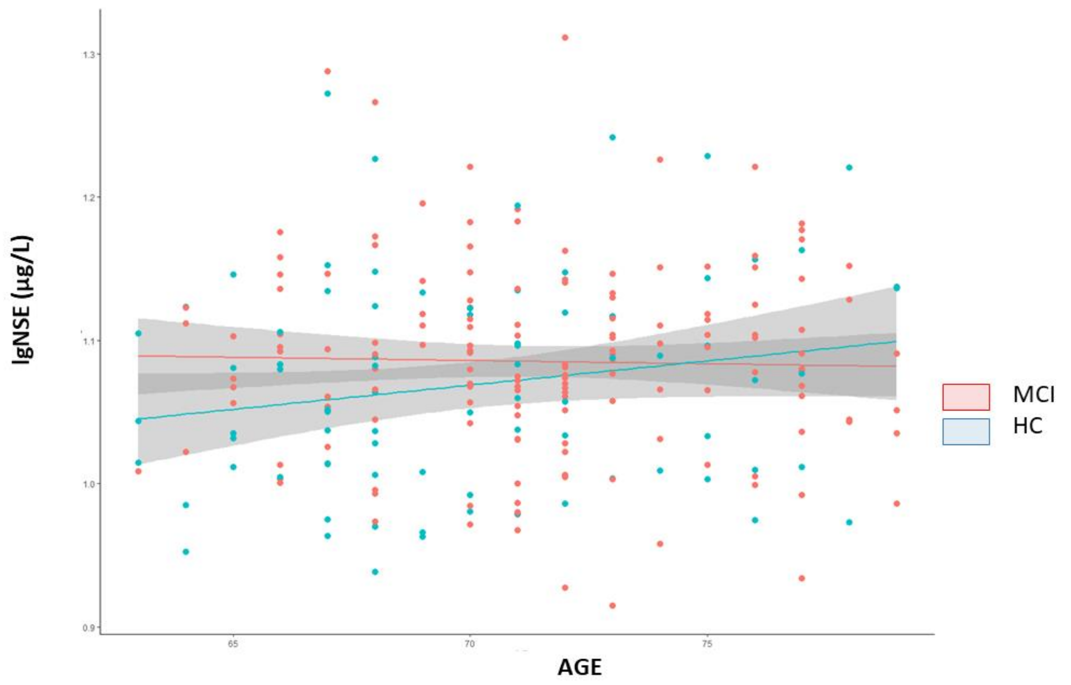

**Supplementary Figure 3 Serum BDNF in mild NCD and HC**

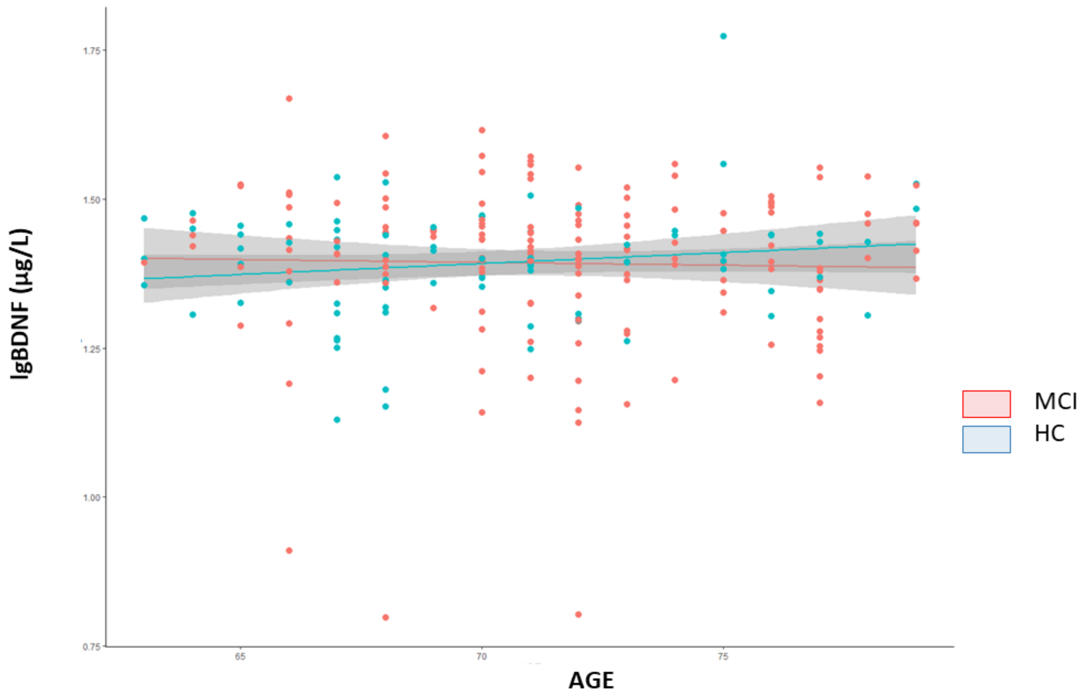

Supplement: Supplementary file 1 [file Data_Sheet_1.PDF]
